# Unveiling the controversy on egg freezing in The Netherlands: A Q-methodology study on women’s viewpoints

**DOI:** 10.1016/j.rbms.2020.09.009

**Published:** 2020-11-09

**Authors:** Johanna Kostenzer, Annelies M.E. Bos, Antoinette de Bont, Job van Exel

**Affiliations:** a Erasmus University Rotterdam, Erasmus School of Health Policy and Management, Rotterdam, the Netherlands; b University Medical Centre Utrecht, Department of Reproductive Medicine and Gynaecology, Utrecht, the Netherlands; c Erasmus University Rotterdam, Erasmus School of Economics, Rotterdam, the Netherlands

**Keywords:** Egg freezing, Assisted reproductive technology, Controversy, Viewpoints, Q-methodology, The Netherlands

## Abstract

Preserving the option to conceive through egg freezing (oocyte cryopreservation) is surrounded by value conflicts and diverse viewpoints, particularly when non-medical or so-called ‘social’ reasons are involved. The debate is controversial and shaped by normative perceptions of the life course, including concepts regarding reproductive ageing, gender, motherhood and biomedicalization. To unravel the controversy and systematically identify the variety of viewpoints on egg freezing, a Q-methodology study was conducted in The Netherlands between December 2018 and October 2019. Thirty-four women of reproductive age participated in the study. They ranked 40 statements according to their level of agreement, and explained their ranking during follow-up interviews. Data were analysed using by-person factor analysis and interpreted using both quantitative and qualitative data. Four viewpoints, of which the fourth was bipolar, were identified: (1) cautious about egg freezing technology; (2) my body, my choice; (3) egg freezing is unnatural; and (4) have children and have them early. The distinct viewpoints illustrate different prioritizations of values and normative dimensions of biomedical innovations. By knowing more about the prevalent opinions on egg freezing and the surrounding controversy, policy makers and practitioners can make better informed decisions in terms of promoting and providing patient-centred infertility care. The findings furthermore stimulate continuing scholarly work on egg freezing and other innovations in reproductive medicine which may continue to disrupt normative standards.

## Introduction

The introduction of egg freezing (oocyte cryopreservation) is surrounded by value conflicts and different understandings of how to regulate fertility preservation in health care, particularly when non-medical reasons are involved (ASRM, 2018, Bozzaro, 2018, Dondorp and De Wert, 2009, Pennings, 2013). Whereas medical egg freezing to prevent adverse outcomes resulting from, for example, cancer treatment (including radio- and chemotherapy) is considered a legitimate option to preserve reproductive potential, freezing eggs for other reasons – usually referred to as ‘non-medical’ or ‘social’ reasons – is less accepted (Daniluk and Koert, 2016, Wennberg et al., 2015). The ongoing debate includes arguments around reproductive autonomy, commercialization, benefits and risks, and equity in access (Dondorp and De Wert, 2009). Women are faced with difficult choices in a stringent framework of societal norms and biomedical paradigms. These concern, amongst others, reproductive ageing, gender, motherhood and medicalization (Dondorp and De Wert, 2009, Kilic and Goecmen, 2018, Pennings, 2013). In this context, women are not only under pressure from the so-called ‘biological clock’ due to the early loss of fecundity (Kilic and Goecmen, 2018, Pennings, 2013), but are also confronted with the societal norm to become the ‘biological mother of a child of one’s own’ (Dondorp and De Wert, 2009) at some point in their lives. Using technology to lift these pressures may, however, be considered as a way of medicalization, leaving the underlying societal problem unaddressed (Dondorp and De Wert, 2009, Shkedi-Rafid and Hashiloni-Dolev, 2011). However, innovations in assisted reproduction are challenging these normative standards, which makes the investigation of viewpoints on egg freezing an interesting case.

Despite its controversy, addressing age-related infertility is one of the core pillars of modern reproductive medicine and a growing market segment in this regard. Increasing childbearing age is a trend across Europe (Eurostat, 2018) and beyond. It can be related to a variety of reasons: sociocultural changes including the increase in women’s labour participation and education, changing value perceptions and family patterns, economic uncertainty, the rise of effective contraception, and the absence of supportive family policies (Mills et al., 2011). In The Netherlands, where egg freezing is allowed for medical and social indications, the average age of women at childbirth has increased from 27.5 years in 1990 to 29.9 years in 2018 (CBS, 2018, CBS, 2019). Aging and fertility, however, are conflicting factors for women’s reproductive lifespan. Assisted reproductive technology has therefore become an increasingly used means for conception at a later stage in life.

It is known from the literature that conflicting perceptions are reflected in the portrayal of users of cryopreservation in various discourses. By using an ethnographic mixed methods approach, including qualitative analysis of mainstream, scientific and marketing literature as well as participant observation, Martin (2010) explored how potential candidates for egg freezing are presented. According to her research, medical egg freezing candidates are portrayed as the ‘worthy cancer patient’. Healthy women seeking social egg freezing, however, are primarily accompanied by three narratives: ‘(1) They are vulnerable to exploitation, (2) they are putting their own selfish needs ahead of more important priorities, and (3) they are liberated and forward-thinking’ (Martin, 2010). Furthermore, a number of empirical studies have focused on the sociological dimension and driving factors to use egg freezing (Baldwin et al., 2015, Baldwin, 2018, Carroll and Krolokke, 2018, Inhorn et al., 2018a, Inhorn et al., 2018b, Inhorn et al., 2018c, Inhorn et al., 2018d, Kilic and Goecmen, 2018), showing that these portrayals are stereotyping egg freezing and its users and do not consider the vast variety of factors involved (Mertes, 2013). The findings, however, illustrate the controversial nature of egg freezing technology and the normativity surrounding it.

Research has been conducted to explore viewpoints on fertility preservation using qualitative or quantitative methods (De Groot et al., 2016, Hodes-Wertz et al., 2013, Schick et al., 2017, Tozzo et al., 2019, Wennberg et al., 2015, Fauser et al., 2019, Meissner et al., 2016). A study conducted in a German cohort explored the relationship between different sociocultural backgrounds (milieus) and attitudes towards social egg freezing (Schick et al., 2017). Although the study revealed interesting insights into sociocultural backgrounds of those in favour of, against or neutral towards using the technology for non-medical reasons (e.g. acceptance was highest among highly-educated participants and participants aged 36–41 years), it did not focus on exploring the prevailing viewpoints on egg freezing in depth. In a study conducted in The Netherlands, 20 in-depth interviews were conducted with women who were interested in pursuing egg freezing at the time of data collection (which occurred in 2011, shortly after the Dutch law was changed to allow social egg freezing) (de Groot et al., 2016). The findings show that the women’s desire for shared parenthood in the future was a key driver for egg freezing. This strong wish also overruled the women’s concerns regarding potential health risks, costs and limited success rates.

Adding to previous research which provides important insights into the considerations and general opinions of women, this study aims to go one step further by systematically exploring the subjective viewpoints of women of reproductive age with Q-methodology as a mixed methods approach. Researching the different perceptions regarding egg freezing makes an interesting case and provides insights into how biomedical innovations in reproductive medicine are perceived, focusing on underlying social norms and policies. It furthermore sheds light on the importance of citizens’ involvement in ethically sensitive decision-making processes in health care. Having clearer insight into the viewpoints that exist around this topic is also helpful in practice, and can support targeted awareness raising and better informed and effective policy making. It moreover acts as a reference case to identify potential changes in opinions in light of further innovations and emergence of technologies. Controversy around egg freezing is mirrored in the terminology used. For the purpose of this paper, however, the terms ‘medical’ and ‘non-medical’ or ‘social’ egg freezing are used. These terms are most commonly used in societal discourse, which is also the focus of this paper.

### Egg freezing in The Netherlands

In 2011, after almost 2 years of discussions, a majority in the House of Representatives of The Netherlands supported the legalization of egg freezing for medical as well as non-medical or social reasons (Bos et al., 2012: 1). Since then, women have been able to consider and access egg freezing, yet the topic has been subject to frequent discussions, also reflected in media coverage (Algemeen Dagblad, 15 October 2014: ‘Apple and Facebook pay women for egg freezing’; de Volkskrant, 16 January 2016, ‘40 years old and a child wish, is this not too late?’, 30 January 2018: 'A smart girl freezes her eggs in time’).

In The Netherlands, egg freezing is closely regulated and may only be provided by licensed healthcare organizations. Eggs may be harvested until the woman reaches 40 years of age, and may be used until her 50th birthday for in-vitro fertilization (IVF)/intracytoplasmic sperm injection (ICSI) treatment. The procedure (including hormone therapy by injections, and vaginal egg retrieval) takes, on average, 2 weeks and approximately 10 eggs are retrieved per cycle. On average, 20 egg cells are required for a realistic chance to achieve pregnancy. This also means that more than one treatment cycle (on average, two to three cycles) is necessary to obtain an adequate number of eggs. The number of retrieved eggs per cycle and the total number of eggs required varies depending on, amongst other factors, egg quality, woman’s age and ovarian reserve markers. Costs vary between 10,000 and 12,000 Euros, with an average cost of 4000 Euros per cycle, to complete a whole egg freezing trajectory (EMC, 2019, UMCU, 2019, VivaNeo, 2019).

Egg freezing is covered by basic health insurance if medical indications apply (e.g. oncology treatment involving radio- or chemotherapy) (NVOG and KLEM, 2018). In the case of social egg freezing, costs are paid privately, which includes medication, stimulation and puncture, treatment, freezing and storage. However, in some cases, costs might be covered by the employer (e.g. some companies may provide coverage as part of their employees’ benefits package; in particular, women working for international companies informed us of this option during follow-up interviews). The use of frozen eggs later in life (e.g. through IVF/ICSI including all treatment steps) must be paid privately, unless the intended parent(s) meet the requirements set out in the current IVF regulations at the time of usage. The costs then depend on the treatment options (e.g. need for sperm donation, number of consultations, IVF or ICSI etc.) (UMCU, 2020). It therefore needs to be highlighted that the costs of egg freezing only reflect part of the financial burden that women need to consider when they decide to have the procedure. The Dutch Association for Obstetrics and Gynaecology (NVOG), together with the Association for Clinical Embryology (KLEM), stress in their position paper on fertility preservation that having children and starting a family is important for many, improving quality of life. For these and other reasons outlined in the text, NVOG and KLEM (2018) argue for full health insurance coverage for measures needed for fertility preservation (yet focusing on medical indications). The associations’ statements are non-binding recommendations, yet they further spark the general discussion around fertility preservation in The Netherlands.

## Materials and methods

Opinions regarding egg freezing are complex and interlinked. We therefore used a Q-methodology approach (Watts and Stenner, 2012) to explore the diversity of views among women in The Netherlands in a systematic manner. In this mixed qualitative–quantitative approach, participants revealed their subjective opinions by ranking a set of 40 statements about egg freezing, and explained their ranking during a follow-up interview. Shared views were identified using by-person factor analysis of the ranking data, and interpreted and described with support of the qualitative data collected during the interviews. This particular approach has been used previously to explore controversial views in the field of health care in general [e.g. for human papillomavirus vaccination (Patty et al., 2017)], and has also been used to investigate sexual and reproductive health and the role of culture (Dune et al., 2017). Hence, this approach was considered useful to systematically analyse and describe the subjective viewpoints on egg freezing.

### Developing the statement set

Representing the variety of aspects that appear in societal discourse on egg freezing, including the often-controversial statements around this topic, was crucial for this study. Therefore, 91 statements were extracted from scientific literature, medical guidelines, magazine and newspaper articles, advertisements, podcasts, social media groups, online fora, websites, expert talks and a small-scale expert survey (conducted during a conference presentation in November 2018). The statements were loosely structured in several categories to ensure coverage of the most relevant aspects shaping opinions on egg freezing (e.g. notions of age and timing, benefit and harm, biological boundaries, coverage, ethics and morality, justice, biomedicalization, ownership, reproductive autonomy and work–life balance). These dimensions may not be considered a stringent categorical framework, yet were used to summarize the main considerations on egg freezing based on the literature (Dondorp and De Wert, 2009, ESHRE Task Force et al., 2012, Pennings, 2013). Following several rounds of discussion and feedback involving all members of the research team, redundant statements were deleted, and unclear statements were rephrased. To evaluate its comprehensiveness, the remaining set of 43 statements was tested in a first pilot (*n* = 3) at the end of January 2019. Here, the data collection process was tested in terms of instructions given, the general procedure and duration. Pilot study participants then reviewed the statement set and shared their feedback, which was discussed later by the research team. Afterwards, the set was revised again, reduced to 40 statements, and translated into Dutch using professional translation services. This set was tested again (*n* = 8) in February 2019, and further small revisions of the set (e.g. wording) were made but the number of statements remained unchanged. The preliminary statement set was presented and commented upon by peers and at an international symposium (with interdisciplinary experts on egg freezing) in June 2019.

### Collecting the data

Women of reproductive age were invited to participate in the study. They were reached via multiple sources. Women with an interest in egg freezing were recruited during two information evenings at University Medical Centre Utrecht (UMCU, 15 April and 2 July 2019). They were informed about the aim of the study and could submit their contact details for the purpose of participation. Furthermore, patients who had finished the egg freezing trajectory or were in the process were recruited via UMCU. Women with no particular personal interest in egg freezing came to know about the study via targeted outreach and mouth-to-mouth recruitment. To achieve greater variation of the sample, especially regarding the participants’ educational background, four additional women were recruited via a research agency. Overall, 34 women between the ages of 24 and 50 years participated in the study. This reflects a sufficient number considering the nature of Q-methodology as a small sample approach. An overview of the study sample can be found in Table 1.

**Table 1:** Characteristics of the study sample (*n* = 34 females).

Characteristics		*n*	%
Age (years)	21–25	3	9%
	26–30	11	32%
	31–35	6	18%
	36–40	10	29%
	41–45	2	6%
	46–50	2	6%
Education level	High school, vocational training	6	18%
	Higher education (Bachelors, Masters, PhD)	28	82%
Religion	Atheism	16	47%
	Christianity	12	35%
	Islam	3	9%
	Other (spirituality)	3	9%
Nationality	Dutch	26	76%
	European other	4	12%
	Asian (Indonesian, Vietnamese)	2	6%
	North African (Moroccan)	2	6%
Children	Yes	4	12%
	No	30	88%
Relationship status	Single	12	35%
	In a relationship	22	65%
Egg freezing experience	None	27	79%
	Undertaken medical egg freezing	1	3%
	Undertaken social egg freezing	6	18%

**Fig. 1:**
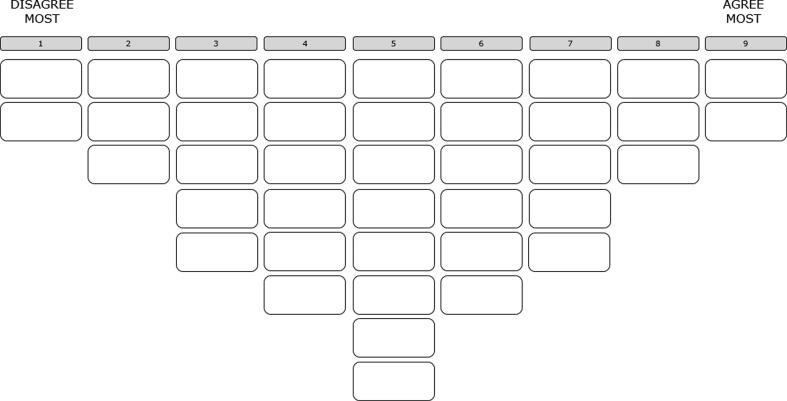
Sorting grid.

Data collection occurred at a place and time suitable to the respondents. The individual interviews were conducted either in Dutch (*n* = 18) or English (*n* = 16). Participants received an information letter before the meeting, and at the beginning of each meeting, they gave informed consent to take part in the study. At all times, they were given the opportunity to ask questions. The respondents were presented with and asked to rank the set of statements on the topic of egg freezing, which were printed on equally sized and styled cards. After dividing the cards into three piles (agree, neutral or do not know, disagree), the participants placed them on a nine-column, forced-choice sorting grid ranging from 1 (‘disagree most’) to 9 (‘agree most’) (Fig. 1). Finally, and after inspecting and making any final adjustments to their ranking, they were asked to explain their choices during a follow-up interview, lasting an average of 20 min, which was recorded and later transcribed. The placing of the statements and the follow-up interview lasted an average of 1 h. Data collection occurred between May and September 2019.

### Analysing the data

We used by-person factor analysis to identify views on egg freezing among women in The Netherlands. This means that, first, correlations were computed between the rankings of the statements by participants and, next, clusters of participants with mutually highly correlated rankings were identified. These clusters, or technically, factors, were then interpreted as viewpoints on egg freezing. The assumption behind this analysis is that participants who rank the statements in a similar way have a similar viewpoint on egg freezing.

The number of factors – and hence viewpoints – to retain and interpret after the analysis was based on the following three criteria (Watts and Stenner, 2012). First, the eigenvalue of factors needed to be >1, meaning that the factor, on average, explains more of the variance in the ranking data than a single participant. Second, at least two participants had to be significantly (*P* < 0.05) associated with each factor, emphasizing that it represents a shared viewpoint. After applying these two statistical criteria, multiple solutions in terms of the number of factors remained; in this case, a maximum of five factors. Therefore, finally, an initial interpretation of the factors in each possible solution was inspected, using both the quantitative data and the qualitative data from the interviews with participants. The solution that provided the most intelligible and comprehensive representation of the data was selected. Here, the solution with four factors was retained.

For each factor in this solution, a weighted average ranking of the statements was computed, called the ‘factor array’. This represents how someone holding that viewpoint on egg freezing (i.e. a hypothetical participant with a correlation of 1 with that factor) would have ranked the statements. These factor arrays were then interpreted and described as the viewpoints on egg freezing, also using the qualitative data collected from participants. A first interpretation was drafted based on the factor arrays, paying particular attention to the characterizing statements (i.e. those that hold the positions −4, −3, + 3 and +4 in the factor array), the distinguishing statements (i.e. those ranked significantly differently in the factor compared with the other factors) and the consensus statements (ranked similarly across factors) for each factor (see Table 2). Next, quotes from the follow-up interviews of respondents loading on a specific factor were used to illustrate and further explain the interpretation of that factor.

**Table 2:** Factor scores per statement.

#	Statement	Viewpoint 1	Viewpoint 2	Viewpoint 3	Viewpoint 4
1	Egg freezing for social reasons stimulates women to postpone childbearing	+1	−2	+1	−1
2	There should be strict age limits for assisted reproduction	−1	−1	0	−1
3	When women want to have children, they should do so at a younger age	−1	−3^a^	−1	+3^a^
4	Women wait too long before starting a family	−1	−3	−1	+2^a^
5	The potential benefits are worth the burden of treatment (hormone therapy and egg cell retrieval)	0	+1^a^	0	−1
6	Although the chance of success is uncertain, it is important that the option is available	+1	+2	+2	+1
7	Egg freezing for social reasons creates false hope about the ability to have children at a later age	0	−2^a^	+2	+1
8	Freezing eggs is preferable over freezing embryos	0	+1	+1	−3^a^
9	Women are insufficiently aware of their fertility lifespan	+3^a^	0	+1	+1
10	Egg freezing for social reasons is a business of hope	+1	−1	+1	−1
11	It is unnatural to preserve fertility beyond the fertile age	+3	−1^a^	+4^a^	+2
12	Just because the option of freezing eggs is available does not mean it should be done	+2^a^	−3	0^a^	−2
13	Egg freezing for medical reasons should be covered by health insurance	+3	+2	+3	+1^a^
14	Egg freezing for social reasons should be covered by health insurance	0	+1^a^	−3^a^	−1
15	Egg freezing for social reasons should be paid for by the user	−1	0	+4^a^	+2^a^
16	Egg freezing for social reasons should be paid for by the employer	−2	−1	−3	0
17	The extension of fertility improves gender equality	0^a^	+2^a^	−2	−3
18	Egg freezing for social reasons promotes equal opportunities for women and men	−2	+3^a^	−1	−4
19	There is insufficient attention for the ethical aspects of egg freezing in general	+2	0	+2	+1
20	Egg freezing for social reasons requires a societal debate	−1^a^	+1^a^	+2^a^	−2^a^
21	I find egg freezing for medical reasons more acceptable than for social reasons	0^a^	−2^a^	+3	+3
22	Not all social reasons for egg freezing are equally good reasons	+1^a^	0^a^	+3^a^	−2^a^
23	Egg freezing is against my convictions	−4	−4	0^a^	+2^a^
24	Starting a family is an ultimate wish of human beings	−3^a^	0	−1	+3^a^
25	Egg freezing for social reasons should be prohibited by law	−2	−4^a^	−3	0^a^
26	People have a right to have a genetic child	+2	+2	−2^a^	+4^a^
27	People have a right to have a child	+4	+4	−2^a^	+4
28	Egg freezing should be available to all women	+2	+3^a^	−1^a^	0
29	Women should be able to freeze their eggs for any reason	−1	+2^a^	−1	0
30	Age-related fertility loss is a medical problem	−3	0	−4	0
31	Women should make unused eggs available for donation	−4	−1^a^	−4	+2^a^
32	Unused eggs should remain in possession of the woman	+4^a^	+1	0	−2^a^
33	Women should be able to make their own choices regarding fertility preservation	+2^a^	+4^a^	0	−2
34	Egg freezing for social reasons allows women to organize their lives without pressure from the ‘biological clock’	−2	+3^a^	0^a^	−2
35	If infertile women want to have children, they should opt for adoption	−3	−2	−2	+1^a^
36	If women take the risk of waiting too long, they should accept the possible consequence of childlessness	+1	−1^a^	+1	0
37	It is difficult to invest in career and family at the same time	+1	+1	0	−4^a^
38	Women should think of their career first and parenthood next	−2	−2	−2	−1
39	Political measures are needed to facilitate parenthood at a younger age	0	0	+1^a^	−1
40	Employers should facilitate parenthood at a younger age	0	0	+2^a^	−3^a^

Statements which are characterizing for a factor are those scoring −4, −3, +3 and +4.

a Distinguishing statements.

The analysis was conducted using PQMethod software (Schmolck and Atkinson, 2018).

### Ethics

The study protocol was approved by the Medical Ethics Committee of Erasmus Medical Centre of Rotterdam (MEC-2018-046) and by the Medical Ethics Committee of University Medical Centre Utrecht (Ref. 19-448/C).

## Results

The quantitative and qualitative analysis of the 34 rankings of the statement set resulted in a four-factor solution, with four distinct viewpoints on egg freezing (see Table 3). However, Viewpoint 4 was defined by two respondents with fairly strong correlations with the factor, one positively and one negatively, making it bipolar. Hence, this viewpoint could be interpreted in two opposing ways, in fact distinguishing a fifth viewpoint. However, as this viewpoint would approximate the reverse of Viewpoint 4, we have discussed one of the interpretations in detail and only highlighted the main characteristics of the contrasting interpretation.

**Table 3:** Factor loadings (*n* = 34).

Respondent	Viewpoint 1 Cautious about egg freezing technology	Viewpoint 2 My body, my choice	Viewpoint 3 Egg freezing is unnatural	Viewpoint 4 Have children and have them early
1	0.2343	0.7011X	0.1648	−0.3447
2	0.4525	0.3712	0.4584	−0.2075
3	0.1502	0.7179X	0.1014	−0.1651
4	0.2817	0.8036X	0.0954	0.1697
5	0.4433X	0.3566	0.1556	−0.0127
6	0.4825	0.1842	0.5482X	−0.0771
7	0.7176X	0.2779	0.2685	0.1088
8	−0.0419	0.8482X	0.0143	−0.1154
9	0.8202X	0.0426	0.0949	0.0598
10	0.4775X	0.1478	0.0984	−0.0615
11	0.0349	0.4063X	0.3076	−0.0573
12	0.2628	0.9036X	−0.1501	−0.0150
13	0.2359	0.3791	0.3312	−0.5596X
14	0.1209	0.8031X	−0.0587	0.1174
15	0.4225	0.5746X	−0.0834	−0.0986
16	0.3393	0.2241	0.6159X	0.0492
17	0.6159X	0.0622	0.3444	0.1531
18	0.2905	0.1555	0.5357	−0.4421
19	0.3496	0.3128	0.4710	0.2302
20	0.3507	0.8268X	−0.2132	0.0321
21	0.0008	0.6323X	0.0909	0.0660
22	0.3869	0.5019	0.3573	−0.0322
23	0.3760	0.6715X	0.1645	0.2291
24	0.1779	0.7307X	0.0171	0.1826
25	0.3377	0.3894	0.1436	0.4430
26	0.4832	0.6251X	0.1947	−0.0025
27	0.5999X	0.1806	0.5220	0.0678
28	0.2301	0.7849X	−0.0618	0.2195
29	0.0752	0.6219X	0.2964	0.3165
30	0.4495	0.6947X	−0.1142	−0.1884
31	0.0495	−0.1252	0.5175X	0.0017
32	0.1903	−0.4782	0.6677X	−0.0980
33	0.0734	−0.1350	0.7366X	0.1480
34	0.1671	0.2314	0.1592	0.5593X

The results have been presented by highlighting the main characterizing and distinguishing statements of each viewpoint. Quotes of the follow-up interviews are used to support the interpretation and share examples of the respondents’ viewpoints.

### Viewpoint 1. Cautious about egg freezing technology

Viewpoint 1 is characterized by strong agreement with the right to have a child (#27, +4; #26, +2):

I think in general everybody has the right to have a child. Then another discussion is about what to do if that is not possible… [Respondent 9]

Starting a family, however, is not considered to be an ultimate wish of human beings (#24, −3).

Respondents defining this view believe that women lack knowledge about their fertile lifespan (#9, +3):

I feel very strongly that people do not know when they are fertile, and some people are uninformed about the stage of their body while it progresses. [Respondent 27]

Still, they also think that women should be able to make own choices regarding their fertility (#33, +2), and having ownership over retrieved egg cells is hence crucial (#32, +4; #31, −4):

I think it's your own body, so you have to be able to make your own decisions about it and that includes fertility. [Respondent 7]

What further defines this viewpoint is the perception that preserving fertility beyond the fertile age is unnatural (#11, +3). Age-related fertility loss is hence not believed to be a medical problem (#30, −3):

I think we should not medicalize things that are not medical. I think this is not medical because it is the natural progression of the bodily state. (…) the loss of fertility is something that has developed through evolution, and I think that it has a purpose that after a certain age this physical mass that you call yourself, that it is not the most optimal anymore to carry children. [Respondent 27]

Nevertheless, respondents with this viewpoint are in favour of cost coverage of egg freezing by health insurance, but only when it is for medical reasons (#13, +3).

Respondents with this viewpoint do not generally oppose egg freezing based on their convictions (#23, −4). However, they stress the need for an ethical debate (#19, +2):

My conviction is mainly science. And I do agree that not everything possible should be done, and that we should really consider the ethical and social implications of the technologies that are available, but I think that this is not something that is forbidden by any morals or principles. [Respondent 27]

(…) it’s also really interesting and cool that there are these kinds of developments, but we should also think about as a whole society how to use them. [Respondent 9]

Ethical concerns furthermore play a role when it comes to employer involvement, as cost coverage of social egg freezing by the employer is seen as critical (#16, −2):

I think it would be wrong if employers could tell you like ‘Oh maybe you can work a bit harder and then in the end we will pay for your egg freezing if it becomes difficult to have a child’. (…) It’s not like you can trade-off having a child later, I think it’s not something we should negotiate about with an employer. [Respondent 17]

This also relates back to the perception of fertility preservation as being unnatural, and supporting cost coverage of medical egg freezing alone.

### Viewpoint 2. My body, my choice

Viewpoint 2 is characterized by emphasis on the freedom to make one’s own choices regarding fertility preservation (#33, +4):

I am strongly in favour of having a choice over my own body – as it is mine. [Respondent 12]

I think it’s important that as a human and as a woman you get to decide what you do with your body and that it’s your choice. [Respondent 4]

In addition, the right to have a child was highly valued in this context (#27, +4):

…because I think it’s kind of a human right actually. You have the right to have a child and it’s still your own choice if you want that or not. [Respondent 12]

Contrary to the other viewpoints, egg freezing is further considered a promotor of equal opportunities for women and men (#18, +3; #17, +2), and a tool to lift the pressure from the ‘biological clock’ (#34, +3):

If you look at how women and men organize their lives and then you look at starting a family, working life, and also finding a relationship or a partner. For women, one out of three is just gone at some point. And I think that if you choose the option to postpone fertility for a while, you can make it more equal. (…) I think that this is all one long process for men, and for women it's divided into parts, so to speak. The period in which you can have children – and the rest of your life. So I think with that you can stretch the fertility of women, just as men have their fertility until later in life. [Respondent 24]

Women with this viewpoint further agree that egg freezing should be available to all women (#28, +3), and hence strongly disagree with legal prohibition of social egg freezing (#25, −4):

I think banning it would be degrading. [Respondent 8]

I think it’s ridiculous that it gets to be forbidden because then it’s against the right to do whatever the heck you want to do with your body. [Respondent 4]

Statements indicating what women ‘should do’ (e.g. have children at younger age) are generally opposed (#3, −3; #35, −2; #38, −2), supporting the importance of the freedom to make autonomous choices as central to this viewpoint. Women further do not believe that egg freezing conflicts with their convictions (#23, −4):

There’s nothing in my convictions, norms and values that would make me think egg freezing is unethical. For me, an egg cell is not yet fertilized life, and even if it was… [Respondent 20]

Interestingly, and contrary to the other views, this viewpoint shows no preference for medical over social reasons (#21, −2), and a positive attitude towards insurance coverage for both indications (#13, +2; #14, +1):

I, for example, find it strange that a differentiation is being made between medical and social reasons. Anyway, I think both should be possible and reimbursed. [Respondent 20]

### Viewpoint 3. Egg freezing is unnatural

In contrast to the first two viewpoints, respondents with Viewpoint 3 share a very critical view towards egg freezing, particularly when social reasons are involved. Participants strongly agree that preserving fertility beyond the fertile age is unnatural (#11, +4):

It’s not for nothing that the body has a certain expiration date so to say, but we as humans think that we always need to work around that. [Respondent 32]

There is a reason given by nature to have kids at a young age. [Respondent 33]

Women with this viewpoint believe that social egg freezing creates false hope about the ability to have children later (#7, +2):

Because it is not just the egg that is needed for the development of the child, it is also the rest of your body that has to work. So you can freeze eggs, but if the nest so to say is not good, and you have to do all kinds of stuff to make it work, then this is also against nature. [Respondent 32]

In general, these respondents do not believe that there is a right to have a child (#26, −2; #27, −2):

No, it’s a gift. And a duty, but certainly not a right. [Respondent 32]

Age-related fertility loss is furthermore not seen as a medical problem (#30, −4), and medical reasons are considered more acceptable than social reasons (#21, +3). Respondents agree that medical egg freezing should be covered by health insurance (#13, +3), whereas social egg freezing should be paid for by the user (#15, +4). Coverage of social egg freezing either by health insurance or the employer is hence strongly opposed (#14, −3; #16, −3):

It’s the woman’s own choice, and she has to pay for it. [Respondent 33]

Yet more than other viewpoints, these respondents see a role for the employer in facilitating parenthood at a younger age (#40, +2).

Respondents with this viewpoint, however, do emphasize autonomous choices regarding unused frozen eggs, and disagree with donating them (#31, −4):

I do not think that you can make this obligatory, because it is something that is not comparable to organ donation. It is something of your own body after all, and you have the right to self-determination, even if it is already stored somewhere. [Respondent 16]

Finally, although respondents with this viewpoint do not think that social egg freezing should be legally banned (#25, −3), they agree that not all social reasons are equally good ones (#22, +3):

If you want to go for a career, then this means that you can’t have kids, you cannot have it all at the same time. [Respondent 32]

Women with this viewpoint hence emphasize the need for a societal debate (#20, +2):

I think that freezing eggs is not only a personal choice, but also a societal development and a trend that we find important in our society. I think that we cannot only talk about individuals who have a wish but also need to talk about the societal issue. [Respondent 31]

### Viewpoint 4. Have children and have them early

Viewpoint 4 is characterized by strong agreement with the right to have a (biological) child (#26, +4; #27, +4), and starting a family is considered an ultimate wish of human beings (#24, +3). Despite some similarities with Viewpoint 3, the strong emphasis on having children at a younger age (#3, +3) and the view that women tend to wait too long to start a family distinguishes it from the other viewpoints (#4, +2).

Preserving fertility after the fertile lifespan is considered unnatural (#11, +2), and also not preferable over freezing embryos (#8, −3), as egg freezing conflicts with the convictions of respondents with this viewpoint (#23, +2):

Maybe you are then just not so healthy anymore or in the ideal state to carry a child and give birth. (…) I just think it’s unnatural, because if your body gives up at a certain moment, ‘now it’s enough, you’re not fertile anymore’, then I think your time is just up. [Respondent 34]

Despite this perceived unnaturalness, medical egg freezing is considered more acceptable than social egg freezing (#21, +3), which is also in line with the positive attitude towards insurance coverage of medical egg freezing (#13, +1):

You are sick because of a certain illness or trauma and it is not your own choice that you can't have a child. But this is medical, you really can't do anything about it. I think that you should then get the chance to freeze and that it is covered by health insurance because it is medical. [Respondent 34]

Social egg freezing, however, should be paid for by the user (#15, +2):

When it's all paid for by the insurance company, then everyone will freeze all their eggs and at a certain moment it will become a kind of ‘oh yeah, I want to work for 30 years first and only then start with children’. Well, I’m very much against that. [Respondent 34]

Investing in family and career at the same time is not perceived as a difficulty (#37, −4), but as an issue of prioritization and planning:

It is your choice whether you go for career or for family. And when you say ‘I'm going for my career now, I'm really busy so I freeze it in’ this shouldn’t work. (…) I think it should just go in a natural way and if you think ‘Oh, I want a career and I want children’ then you need to be able to plan that well. [Respondent 34]

The employer is therefore not considered to carry responsibility for facilitating parenthood at an earlier age (#40, −3) or for paying for egg freezing (#16, 0).

Finally, this viewpoint is characterized by a clear disapproval of positive effects of egg freezing on gender equality and equal chances between women and men (#17, −3; #18, −4). Respondents with this viewpoint do not agree that women should be able to make their own choices about fertility preservation (#33, −2). In line with this, and quite contrary to other viewpoints, they feel that unused eggs do not need to remain in the possession of the woman (#32, −2), and should rather be made available for donation (#32, +2).

To note here, and as mentioned before, Viewpoint 4 is defined by two respondents with fairly strong correlations with the factor, one positively and one negatively, making it bipolar. This means that there is also a second, approximate inverse interpretation of this factor. This contrasting viewpoint on egg freezing is characterized by a positive attitude to egg freezing for medical reasons, as a way for women to make their own choices about fertility preservation and giving them more equal chances to invest in both a career and a family:

For men it [having children] advances their career and for women it doesn’t. So I think once women have the opportunity to have children later on…it would take away some of those inequalities. [Respondent 13]

## Discussion

Egg freezing is a specific field of reproductive medicine that is surrounded by controversial debate and shaped by normative perceptions of the life course, including concepts regarding reproductive ageing, gender, motherhood and biomedicalization, as outlined previously (Dondorp and De Wert, 2009, Pennings, 2013). This research reveals new insights into how arguments related to these concepts are perceived by women in The Netherlands.

Several studies have been conducted on women’s motivations, fertility intentions and opinions. This study adds to the existing scholarly work by investigating the subjective viewpoints among women of reproductive age regarding egg freezing in a systematic manner using Q-methodology.

The diversity of values at stake and the linked controversies are reflected in the four distinct perspectives identified in this research. The first viewpoint (cautious about egg freezing technology) is characterized by a permissive attitude towards the use of egg freezing in general, yet highlighting the importance of conscious and careful use, and the need for an ethical debate. The second viewpoint (my body, my choice) takes a liberal feminist perspective, emphasizing potential positive effects in terms of gender equality, and valuing autonomy and ownership over one’s own body. The third viewpoint (egg freezing is unnatural) stresses the artificial nature of using a technology for future conception, considering age-related fertility loss a biological process which should not be interfered with using technology. The fourth and final viewpoint (have children and have them early) is characterized by a more traditional perception of motherhood that emphasizes the merits of having children at a younger age, hence disapproving of medical intervention unless clearly defined medical reasons are involved.

This study adds to our understanding of the prevailing normative concept of biomedicalization by discussing arguments around health and disease, commercialization and equity in access. Particularly reflected in Viewpoints 3 and 4, a clear prioritization of medical reasons over social reasons was identified. This is expressed in stronger acceptance of medical egg freezing, and insurance coverage of medical egg freezing but not social egg freezing (#13–16). Reasons brought forward to explain the prioritization of medical over social reasons were the potential elective nature of social egg freezing, and the absence of a disease and related lack of control. Women holding these viewpoints opposing egg freezing for social reasons nevertheless showed a certain empathy towards women suffering from a certain medical condition. Women seeking social egg freezing, however, were perceived to have other options. This observation relates to the research conducted by Martin (2010) which found that medical egg freezers are considered as ‘worthy cancer patients’ and receive more empathy than candidates for social egg freezing. Contrary to this, however, and reflected in Viewpoint 2, is the less stringent differentiation into medical and social egg freezing, and a more positive attitude towards insurance coverage of both indications, which is in line with the liberal stance this viewpoint takes.

This study further identified interesting insights regarding perceptions of the life course, reproductive timing and the perceived ideal conditions in this respect. Despite not creating strong reactions in terms of ranking the statements, responses which were triggered by Statements #37 (It is difficult to invest in career and family at the same time) and #39 (Political measures are needed to facilitate parenthood at a younger age) are considered worthy of sharing. Irrespective of their level of agreement, during the follow-up interviews, participants holding Viewpoints 1–3 highlighted the difficulty of having the right conditions for starting a family. It was mentioned that this, however, was not solely related to employment conditions not permitting earlier childbearing, and was also due to conditions experienced in (higher) education and training, expensive housing etc. These experienced pressures were considered not only to promote the postponement of childbearing in general, but also of building and sustaining stable relationships. The particular wish of women for ‘shared parenthood’, as identified in the study conducted by de Groot et al. (2016) in The Netherlands, provides context-specific explanations for these pressures experienced in a society striving for gender equality. These findings also relate to previous research on women’s motivations and considerations explored in various countries (e.g. UK, Turkey, USA and Israel) (Baldwin et al., 2015, Baldwin, 2018, Carroll and Krolokke, 2018, Inhorn et al., 2018a, Inhorn et al., 2018b, Inhorn et al., 2018c, Inhorn et al., 2018d, Kilic and Goecmen, 2018). Results show that uncertain living conditions (e.g. lacking financial resources, job insecurity) and relationship factors (e.g. singlehood and instable relationships), rather than women’s career ambitions, are the main reasons for postponing childbearing and/or seeking non-medical egg freezing. What we can further learn from this is that perceived societal norms seem to be in conflict with the realities that women face, contributing towards the gap between the ideal biological time and the realized time for childbearing.

This study, however, also has limitations which need to be considered. Due to the sensitivity of the topic, we faced difficulties in generating a more diverse sample. Despite taking necessary measures and also recruiting women via a research agency, highly educated women and women without children are somewhat over-represented in the study. However, we expect the opinions identified here to represent women’s central thoughts and struggles regarding this topic. Furthermore, this study does not provide information about the distribution of these viewpoints among women in The Netherlands. In Q-methodology, however, the focus is on the comprehensiveness of the statement set and generalizability to the subject matter, not on the respondent sample. Information about the distribution of the viewpoints and relations to characteristics and wider beliefs of women can be investigated using survey techniques (Baker et al., 2010). Finally, Viewpoint 4 is defined by two respondents with fairly strong correlations with the factor, one positively and one negatively, making it a bipolar factor. On the one side, with a positive correlation, a respondent with strong family orientation and a more conservative belief. On the other side, with a negative correlation, a respondent with a more individualistic and libertarian perspective. This factor thus contains two contrasting viewpoints on egg freezing, each representing a coherent and recognizable story. As these viewpoints are approximately opposite, we discussed one in more detail and only highlighted the main characteristics of the contrasting viewpoint. In this study, we focused on women’s viewpoints on egg freezing. We are aware, however, that neither fertility nor reproduction are exclusively female topics. While we think that exploring women’s viewpoints is highly relevant, we suggest that, in order to take a more societal perspective, future research should also include men’s voices in general, and also particular stakeholder groups such as healthcare professionals, policy makers and ethicists. Adding to this, it will be interesting to investigate whether viewpoints on egg freezing differ across countries with different cultural contexts and welfare systems. A similar study is being conducted in Austria for this purpose to explore the controversy in a country where social egg freezing is (currently) not permitted.

## Conclusions

Exploring women’s viewpoints on egg freezing in The Netherlands allowed us to take a closer look at the controversy which surrounds egg freezing and its use. The study identified four distinct viewpoints, and shows that women share yet are also confronted with societal norms and biomedical paradigms which we reflected upon. What is important is that viewpoints – no matter how conflicting they might be – need to be acknowledged to better understand underlying value perceptions and normative dimensions in this regard. This is important to create awareness and understanding for the prevalent viewpoints that exist, and the underlying controversy surrounding them. While the findings of this study can support policy makers and practitioners to make better informed decisions in terms of promoting and providing patient-centred infertility care (Dancet et al., 2011, Inhorn et al., 2018d), they also provide a starting point for a societal discussion of (women’s) perceived pressures regarding childbearing in a broader sense. Finally, the findings stimulate continuing scholarly work on egg freezing and further innovations in reproductive medicine which may continue to disrupt normative standards.

## Declaration

*The authors report no financial or commercial conflicts of interest*.
